# Supplementary material for: NPTX2 and cognitive dysfunction in Alzheimer’s Disease
Source: eLife. 2017 Mar 23;6:e23798. doi: 10.7554/eLife.23798 (PMC5404919; doi:10.7554/eLife.23798)
Supplement: Figure 1—source data 1. — DOI: http://dx.doi.org/10.7554/eLife.23798.003 [file elife-23798-fig1-data1.docx]

**Figure 1 – source data 1. Clinical and histopathological information of ASYMAD and AD individuals for brain analysis.**

| # | Clinical diagnosis | CERAD | BRAAK | Age | Sex | Race | PMD  (hours) | Education | Final MMSE |
| --- | --- | --- | --- | --- | --- | --- | --- | --- | --- |
| 1 | Control | N/A | N/A | 69 | F | W | 13 | N/A | N/A |
| 2 | Control | N/A | N/A | 83 | M | W | 21 | N/A | N/A |
| 3 | Control | N/A | N/A | 86 | M | W | 21 | N/A | N/A |
| 4 | Control | N/A | N/A | 71 | M | W | 14 | N/A | N/A |
| 5 | Control | N/A | N/A | 80 | F | W | 66 | N/A | N/A |
| 6 | Control | N/A | N/A | 72 | M | W | 24 | N/A | N/A |
| 7 | Control | N/A | N/A | 71 | M | B | 16 | N/A | N/A |
| 8 | Control | N/A | N/A | 79 | F | W | 24 | N/A | N/A |
| 9 | Control | N/A | 1 | 80 | F | W | 6 | N/A | N/A |
| 10 | Control | N/A | N/A | 88 | M | W | 10 | N/A | N/A |
| 11 | Control | N/A | N/A | 73 | M | W | 9 | N/A | N/A |
| 12 | Control | N/A | N/A | 83 | F | W | 8 | N/A | N/A |
| 13 | Control | N/A | 1 | 79 | M | W | 10 | N/A | 29 |
| 14 | Control | N/A | N/A | 59 | M | W | 12 | N/A | N/A |
| 15 | Control | 0 | 3 | 80 | M | W | 22 | N/A | N/A |
| 16 | Control | A | 3 | 94 | M | W | 16 | 20 | 30 |
| 17 | Control | N/A | 1 | 91 | F | W | 8 | N/A | N/A |
| 18 | Control | 0 | 4 | 99 | M | B | 24 | 22 | 28 |
| 19 | Control | 0 | 4 | 86 | M | W | 7 | 18 | 29 |
| 20 | Control | 0 | 3 | 95 | M | W | 17 | 9 | 26 |
| 21 | AD | N/A | N/A | 83 | F | W | 18 | N/A | N/A |
| 22 | AD | N/A | N/A | 81 | M | W | 7 | 18 | 19 |
| 23 | AD | B | 5 | 96 | M | W | N/A | N/A | N/A |
| 24 | AD | N/A | N/A | 86 | M | W | 8 | 25 | N/A |
| 25 | AD | C | 4 | 98 | M | W | 20 | 20 | N/A |
| 26 | AD | C | 4 | 92 | M | W | 7 | 20 | N/A |
| 27 | AD | C | 6 | 87 | F | W | 17.5 | N/A | N/A |
| 28 | AD | C | 6 | 80 | F | W | 6.5 | N/A | N/A |
| 29 | AD | C | 6 | 83 | F | W | 15.5 | N/A | N/A |
| 30 | AD | C | 6 | 87 | F | B | 16 | N/A | N/A |
| 31 | AD | C | 6 | 85 | M | W | 3.5 | N/A | N/A |
| 32 | AD | C | 5 | 79 | M | W | 10.5 | N/A | N/A |
| 33 | AD | C | 5 | 84 | F | W | 5 | N/A | N/A |
| 34 | AD | C | 6 | 72 | F | W | 19 | N/A | N/A |
| 35 | AD | C | 6 | 92 | F | W | 13 | 18 | 14 |
| 36 | AD | C | 6 | 92 | M | W | 12 | 16 | 4 |
| 37 | AD | C | 6 | 82 | M | W | 23 | 18 | 3 |
| 38 | AD | C | 6 | 83 | F | W | 11 | 12 | N/A |
| 39 | AD | C | 6 | 94 | F | W | 17.5 | 14 | N/A |
| 40 | AD | C | 6 | 85 | F | W | 18 | N/A | N/A |
| 41 | AD | C | 6 | 79 | F | W | 4 | N/A | N/A |
| 42 | AD | C | 6 | 58 | F | W | 8.5 | N/A | N/A |
| 43 | AD | C | 4 | 85 | M | W | 6 | N/A | N/A |
| 44 | AD | C | 5 | 84 | M | W | 5 | N/A | N/A |
| 45 | AD | C | 5 | 86 | M | W | 5 | N/A | N/A |
| 46 | AD | C | 5 | 90 | F | W | 5 | N/A | N/A |
| 47 | AD | C | 5 | 62 | M |  | 13 | N/A | N/A |
| 48 | AD | B | 6 | 83 | F | W | 4 | N/A | N/A |
| 49 | AD | C | 6 | 73 | F | W | 6 | N/A | N/A |
| 50 | AD | C | 6 | 83 | F | W | 3 | N/A | N/A |
| 51 | ASYMAD | B | 2 | 85 | F | W | 17 | N/A | 29 |
| 52 | ASYMAD | B | 4 | 95 | F | W | 2 | 17 | 28 |
| 53 | ASYMAD | B | 4 | 92 | M | W | 18 | 20 | 28 |
| 54 | ASYMAD | C | 4 | 75 | M | W | 24 | 16 | 29 |
| 55 | ASYMAD | B | 4 | 92 | F | W | 18 | 13 | 30 |
| 56 | ASYMAD | B | 4 | 92 | F | W | 12 | N/A | N/A |
| 57 | ASYMAD | B | 4 | 83 | M | W | 6 | N/A | N/A |
| 58 | ASYMAD | B | 3 | 96 | M | W | 5.5 | N/A | N/A |
| 59 | ASYMAD | B | 3 | 96 | M | W | 10.5 | N/A | N/A |
| 60 | ASYMAD | B | 4 | 86 | F | W | 2.5 | N/A | N/A |

PMD: postmortem delay.
