# Supplementary material for: NPTX2 and cognitive dysfunction in Alzheimer’s Disease
Source: eLife. 2017 Mar 23;6:e23798. doi: 10.7554/eLife.23798 (PMC5404919; doi:10.7554/eLife.23798)
Supplement: Figure 1—source data 2. — DOI: http://dx.doi.org/10.7554/eLife.23798.004 [file elife-23798-fig1-data2.docx]

**Figure 1 – source data 2. Information of individuals with Down syndrome for brain analysis.**

| # | Clinical diagnosis | Age | Sex | PMD (hours) |
| --- | --- | --- | --- | --- |
| 1 | Control | 19 | M | 14 |
| 2 | Control | 22 | M | 8 |
| 3 | Control | 23 | M | 8 |
| 4 | Control | 25 | M | 23 |
| 5 | Control | 39 | F | 17 |
| 6 | Control | 40 | M | 17 |
| 7 | DS | 19 | M | 26 |
| 8 | DS | 22 | M | 15 |
| 9 | DS | 23 | M | 24 |
| 10 | DS | 25 | M | 24 |
| 11 | DS | 39 | F | 12 |
| 12 | DS | 40 | M | 10 |

PMD: postmortem delay.
