# Supplementary material for: NPTX2 and cognitive dysfunction in Alzheimer’s Disease
Source: eLife. 2017 Mar 23;6:e23798. doi: 10.7554/eLife.23798 (PMC5404919; doi:10.7554/eLife.23798)
Supplement: Figure 1—source data 3. — DOI: http://dx.doi.org/10.7554/eLife.23798.005 [file elife-23798-fig1-data3.docx]

**Figure 1 – source data 3. Information of individuals with Down syndrome and Alzheimer’s disease for brain analysis.**

| # | Clinical diagnosis | CERAD | BRAAK | Age | Sex |
| --- | --- | --- | --- | --- | --- |
| 1 | Control | N/A | N/A | 86 | M |
| 2 | Control | N/A | N/A | 92 | F |
| 3 | Control | 0 | 0 | 31 | M |
| 4 | Control | 0 | 0 | 61 | F |
| 5 | Control | A | 0 | 59 | F |
| 6 | Control | 0 | 0 | 78 | M |
| 7 | Control | 0 | 0 | 68 | F |
| 8 | Control | N/A | N/A | 71 | M |
| 9 | DS-AD | C | VI | 58 | F |
| 10 | DS-AD | C | IV | 43 | F |
| 11 | DS-AD | C | V | 54 | M |
| 12 | DS-AD | C | VI | 59 | F |
| 13 | DS-AD | C | VI | 59 | M |
| 14 | DS-AD | C | V | 48 | N |
| 15 | DS-AD | C | VI | 57 | F |
| 16 | DS-AD | C | VI | 56 | M |
| 17 | DS-AD | C | VI | 61 | M |
| 18 | DS-AD | C | V | 43 | F |
| 19 | DS-AD | C | VI | 74 | M |
| 20 | DS-AD | C | VI | 63 | M |
