# Supplementary material for: NPTX2 and cognitive dysfunction in Alzheimer’s Disease
Source: eLife. 2017 Mar 23;6:e23798. doi: 10.7554/eLife.23798 (PMC5404919; doi:10.7554/eLife.23798)
Supplement: Figure 4—source data 1. — DOI: http://dx.doi.org/10.7554/eLife.23798.015 [file elife-23798-fig4-data1.docx]

**Figure 4 – source data 1. Information of young healthy controls and aged healthy controls for brain analysis.**

| # | Clinical diagnosis | Age | Sex | Race | PMD (hours) |
| --- | --- | --- | --- | --- | --- |
| 1 | Control | 21 | M | AA | 26 |
| 2 | Control | 22 | M | B | 43 |
| 3 | Control | 22 | M | W | 13 |
| 4 | Control | 23 | M | W | 15 |
| 5 | Control | 23 | M | W | 33 |
| 6 | Control | 24 | M | W | 18 |
| 7 | Control | 25 | M | AA | 28 |
| 8 | Control | 30 | M | W | 17 |
| 9 | Control | 31 | F | AA | 12 |
| 10 | Control | 34 | M | W | 31 |
| 11 | Control | 37 | M | AA | 9 |
| 12 | Control | 37 | M | W | 44 |
| 13 | Control | 79 | F | W | 24 |
| 14 | Control | 80 | F | W | 6 |
| 15 | Control | 88 | M | W | 10 |
| 16 | Control | 73 | M | W | 9 |
| 17 | Control | 79 | M | W | 10 |
| 18 | Control | 59 | M | W | 12 |
| 19 | Control | 91 | F | W | 8 |
| 20 | Control | 71 | F | W | 14 |
| 21 | Control | 80 | F | W | 66 |
| 22 | Control | 71 | M | B | 16 |
| 23 | Control | 95 | M | W | 17 |
| 24 | Control | 69 | F | W | 13 |
| 25 | Control | 83 | M | W | 21 |
| 26 | Control | 86 | M | W | 21 |

PMD: postmortem delay.
