# Supplementary material for: NPTX2 and cognitive dysfunction in Alzheimer’s Disease
Source: eLife. 2017 Mar 23;6:e23798. doi: 10.7554/eLife.23798 (PMC5404919; doi:10.7554/eLife.23798)
Supplement: Figure 5—source data 1. — DOI: http://dx.doi.org/10.7554/eLife.23798.017 [file elife-23798-fig5-data1.docx]

**Figure 5 – source data 1. Summary of human CSF analysis in Alzheimer’s disease (1^st^ cohort).**

|  | Normal control  n = 36 | Alzheimer’s Disease  n = 30 | Control vs AD |
| --- | --- | --- | --- |
| Age (Years) | 70.44 ± 8.16 | 72.97 ± 10.44 | p = 0.27 |
| Education (Years) | 16.72 ± 2.23 | 16.34 ± 2.42 | p = 0.52 |
| Gender (F / M) | 24 / 12 | 11 / 19 |  |
| MMSE | 29.53 ± 0.81 | 23.40 ± 4.45 | p < 0.0001 |
| NPTX2^a^ | 100.00 ± 45.03 | 44.60 ± 39.10 | p < 0.0001 |
| NPTX2^b^ (pg/ml) | 1067.23 ± 521.85 | 295.56 ± 188.89 | p < 0.0001 |
| NPTX1^a^ | 100.00 ± 58.78 | 60.09 ± 32.28 | p = 0.0015 |
| NPTXR^a^ | 100.00 ± 80.10 | 50.29 ± 44.78 | p = 0.0053 |
| ApoE ε4 (%) | 38 | 62 |  |
| Aβ42 (pg/ml) | 238.68 ± 67.70 | 162.82 ± 64.97 | p < 0.0001 |
| p-Tau181 (pg/ml) | 27.83 ± 9.62 | 36.99 ± 15.62 | p = 0.009 |
| Tau (pg/ml) | 68.54 ± 23.04 | 95.20 ± 37.26 | p = 0.002 |

CSF samples were obtained under IRB-approved research protocols, and handled and stored following best practices. Levels of Aβ42, total Tau and p-Tau181 were measured using the Inno AlzBio3 multiplex assay kits, and read on a Bio-Rad X-Map plate reader. MMSE: mini mental state examination. ^a^ NPTXs levels in CSF were assayed by Western blot; ^b^ NPTX2 levels in CSF were measured by ELISA. Data represent mean ± SD.
