# Supplementary material for: NPTX2 and cognitive dysfunction in Alzheimer’s Disease
Source: eLife. 2017 Mar 23;6:e23798. doi: 10.7554/eLife.23798 (PMC5404919; doi:10.7554/eLife.23798)
Supplement: Figure 5—source data 2. — DOI: http://dx.doi.org/10.7554/eLife.23798.018 [file elife-23798-fig5-data2.docx]

**Figure 5 – source data 2. Summary of human CSF analysis in Alzheimer’s disease (2^nd^ cohort).**

|  | Normal control  n = 36 | Alzheimer’s Disease  n = 30 | Control vs AD |
| --- | --- | --- | --- |
| Age (Years) | 72.20 ± 7.78 | 72.24 ± 10.15 | p = 0.99 |
| Gender (F / M) | 22 / 14 | 16 / 14 |  |
| DRS | 140.26 ± 4.05 | 114.63 ± 13.08 | p < 0.0001 |
| MMSE | 29.36 ± 0.87 | 22.00 ± 3.69 | p < 0.0001 |
| NPTX2^a^ | 100.00 ± 48.21 | 62.55 ± 46.71 | p = 0.0022 |
| NPTX2^b^ (pg/ml) | 1112.79 ± 699.36 | 716.12 ± 388.22 | p = 0.0073 |
| NPTX1^a^ | 100.00 ± 45.45 | 67.39 ± 44.32 | p = 0.0046 |
| NPTXR^a^ | 100.00 ± 73.57 | 59.44 ± 55.67 | p = 0.0229 |
| Aβ42 (pg/ml) | 214.10 ± 74.03 | 133.61 ± 53.71 | p < 0.0001 |
| p-Tau181 (pg/ml) | 34.72 ± 19.66 | 47.88 ± 20.52 | p = 0.019 |
| Tau (pg/ml) | 61.64 ± 29.43 | 117.15 ± 74.35 | p = 0.0005 |

CSF samples were obtained under IRB-approved research protocols, and handled and stored following best practices. Levels of Aβ42, total Tau and p-Tau181 were measured using the Inno AlzBio3 multiplex assay kits, and read on a Bio-Rad X-Map plate reader. DRS: dementia rating scale; MMSE: mini mental state examination. ^a^ NPTXs levels in CSF were assayed by Western blot; ^b^ NPTX2 levels in CSF were measured by ELISA. Data represent mean ± SD.
